# Some Surgical Operations Recently Performed in the Macon Hospital*Reported to the Surgical Section of the Macon Medical Society, March 16, 1897.

**Published:** 1897-05

**Authors:** Howard J. Williams

**Affiliations:** Macon, Ga.


### ORIGINAL COMMUNICATIONS.

---

### SOME SURGICAL OPERATIONS RECENTLY PERFORMED IN THE MACON HOSPITAL.*

By HOWARD J. WILLIAMS, A.M., M.D.,

MACON, GA.

The following cases are reported from the records taken at the hospital during the past few months. While one or two are of ordinary interest the others are rather unusual.

CASE 1. *Varicocele. Open-Excision.*—W. W., male, white, age seventeen years, laborer, was admitted to the male charity ward October 15, 1896, suffering with an acute attack of malarial fever. During his convalescence he complained to the resident physician, Dr. W. J. Little, of pain in the left testicle, which was found to be somewhat diminished in size, while the sac was filled by a large bundle of enlarged veins. The patient stated that this condition had existed since childhood, but in recent years it had increased and was now attended by constant dragging pain and discomfort, for which he wished for more relief than was obtained from suspensory bandages. He was transferred to the surgical side, and on October 27, 10 A.M., I operated, assisted by Dr. Little and Dr. Derry, the latter administering ether. The method of open-excision was employed, and the wound dressed with iodoform, iodoform gauze and collodion. After the operation his tem-

*Reported to the Surgical Section of the Macon Medical Society, March 16, 1897.

perature rose to 101.4, but next morning it fell to the normal under the use of quinine. After this he made an uneventful recovery. Primary union having taken place, he was dismissed on the twelfth day.

Open-excision is, as you know, simply cutting down into the scrotum in the line of the veins, opening the sheath of the veins, separating them from the spermatic artery and duct, throwing around the mass of veins two catgut ligatures about one-half to one and a half inches a part, then cutting out the section embraced between the two ligatures and tying the stumps together, the wound being closed either by interrupted or continuous sutures.

I have employed this method since 1891 in preference to subcutaneous ligation and the button operation of Dr. W. H. Pancoast, which had been the operation I had previously employed. By it there is less liability of embolus and septic poisoning. It is certainly less painful and the operation is an ideal one. It has usually been my practice to simply close up the knife wound in the line of the incision—*i. e.*, vertically. In the present case, however, upon the suggestion of Dr. Little, I converted the vertical incision into a transverse wound by stitching the two angles together and closing up the wound on either side. This shortens the scrotum to some extent and gives a greater support to the testicle.

The rise in temperature in the case was simply due to cachexia; we frequently see a rise of temperature in malarial patients follow any surgical operation, which is easily reduced by quinine, and does not complicate the recovery of the case.

I do not think the operation is frequently demanded, and would in any case coming to me for treatment, first suggest the employment of suspensory bandages, reserving the operation for advanced cases accompanied by continuous pain and discomfort, or attended by rapid atrophy of the testicle.

Case 2. *Appendicitis.*—A. N., negro, age sixteen years, laborer, admitted to the male charity ward, September 30, 1896, with fever, pain, tympany, a distinct swelling in the right iliac fossa, and bogginess of the abdominal walls over the mass. One year previous he had chills and fever; in May, 1896, had an attack of pain in the side, vomiting and fever confining him to bed for three weeks. Three weeks prior to admission he had a recurrence which lasted a few days. He then went to work, feeling well he says up to two days before coming into the hospital. He was evidently in the third or fourth attack and had an appendicular abscess. October 1, 10 A.M., assisted by Dr. Little, Dr. Derry, and Dr. Mc Hatton, the former giving ether, I operated, employing a vertical incision over McBurney's point.

On reaching the peritoneum I found it somewhat separated from the overlying tissues by some fluid; opening it the intestines were found matted together and to the peritoneum by large masses of organized lymph which could be separated but slightly from the bowel. The intestine was highly injected, red and angry. It was difficult to decide in which way to go to find the abscess cavity, a drop of pus, however, oozing up from the right side of the wound indicated the direction, and after protecting the wound with gauze packing the opening was gently enlarged. Quite a quantity of pus escaped. The cavity was washed out carefully and a tentative effort made to find the appendix in the bottom of the wound, but without results. A drainage-tube was placed down to the bottom of the cavity and iodoform gauze wicks placed around it. The external wound partially closed and the usual dressings applied. The temperature at the time of the operation was 98.8, pulse 110, respirations 26; following the operation the evening temperature was 98.6, pulse and respiration reduced. He made an uneventful recovery, was dismissed October 27, and as he has not been heard of since, it is concluded he is doing well.

This is the second case in which there was such an extensive exudation of organized lymph in these cases that I saw during the year 1896, and I wish to ask others if they have found it in these cases of recurring attacks of appendicular inflammation? In the other case occurring in a neighboring town, I operated late at night, and after a careful search for pus I had to abandon the investigation (the patient was extremely weak and was showing unpleasant chloroform symptoms), and packing the wound with iodoform gauze wait either for nature to follow the artificial opening I had prepared for her, or later renew my efforts. Fortunately in the next twelve hours the lymph began to break down and the abscess discharged through the wound.

It is dangerous, in my estimation, to attempt to separate these adhesions too much, for an opening may be made in the general peritoneum and pus escape into the abdominal cavity. In these abscesses it is better not to make too vigorous an effort to find the appendix, but simply rely upon incision and drainage.

Dr. Little says he saw this patient two months ago. He was doing well.

CASE 3. *Fracture of the Pelvis, Laceration of the Bladder. Laparotomy, Death after Sixteen Hours.*—A. M., male, white, twenty-two years, railroad postal clerk, at 8 P.M., October 9, 1896, arrived on the Georgia Railroad train, at the Ocmulgee bridge. At this point all trains stop to register before proceeding into the city. The stop is made on a high embankment and a trestle that passes over the Southern Railway tracks, twenty-five feet beneath. For some purpose he stepped off the postal car and fell down to the track of the Southern Railway, striking the pubic bone, it is supposed, on the railroad iron. He was taken up conscious, and carried to the Southern Railway passenger depot a short distance away, and I was summoned. He presented no external evidence of injury whatever, but was suffering with intense shock, severe pain in the lower abdomen, great thirst, and an excruciating desire to urinate; evidences of severe internal injury, concealed hemorrhage, and lesions of the urinary tract.

After a hypodermic of morphia, strychnia, and whiskey, I passed a catheter but could get no urine. I had him removed to the hospital, and having him chlorforomed for examination, I again catheterized him and drew off half a pint of blood. The abdomen was distended, dull on percussion to the right side, tympanitic to the left and above the umbilicus.

Assisted by Dr. McHatton, Dr. Little, and Dr. Derry, the latter administering the chloroform, I proceeded to perform a laparotomy; expecting to find an internal hemorrhage and a laceration either of the kidney or other portion of the urinary system. Blood was found diffused throughout the parietal coverings of the abdomen, the muscles were dissected apart and the peritoneum separated from the overlying muscles by the hemorrhage. On introducing my finger into the incision and passing it downwards to the region of the bladder, I found the pubic bone fractured, comminuted, and the bladder perforated by the roughened end of one of the pieces. The wound in the bladder was about one and a half inches long, partially extraperitoneal and partly intraperitoneal. The wound and abdominal cavity were washed out with the idea of clearing the field to sew up the wounded bladder, but he began to go down so rapidly that we could only put in glass drainage-tubes and pack the cavity around with iodoform gauze and get him into bed. Whiskey, strychnia, nitroglycerine, transfusion of salt solution was used to control the shock and hemorrhage, and morphia to control the pain. This treatment was continued for the next few hours, but he died at 2 P.M., October 10th, 1896, sixteen hours after the accident.

CASE 4. *Stab Wound of the Abdomen. Laparotomy.*—C. H., female, negro, age thirty years, cook, admitted to the woman's charity ward, 9 P.M., March 6, 1897, with a stab wound of the right hypochondriac region, one and a half inches long and penetrating the abdominal cavity, one and a half inches above and two inches to the right of the umbilicus; also an incised wound three inches long and an inch deep on the anterior surface of the thigh. When she was brought to the hospital one hour after the fight the omentum was protruding through the wound. She was in very good condition, shock only slight. Temporary aseptic dressings were immediately applied and I was summoned.

Assisted by Dr. Little and Dr. O. H. Weaver, the latter administering chloroform, I enlarged the abdominal wound to examine the intestines for possible wounds. None were found. The omentum was carefully examined for foreign bodies and thoroughly cleansed. The intestines and omentum were then replaced, and the abdomen closed with silk sutures. For forty-eight hours she was allowed no food, only a little water, then thin chicken broth for two days, followed later by milk and a gradual return to the ordinary diet. She had comparatively little fever, never over 100, making a rapid and uneventful recovery. She was discharged March 26, twenty-one days after admission.

As to the possibility of internal hemorrhage, there was none; the protruding omentum plugged up the wound so as to control the hemorrhage from the arteries of the abdominal walls. While enlarging the wound we encountered a considerable hemorrhage from one of these arteries; owing to the great thickness of the abdominal parietes—the woman was very fat—it was quite difficult to find the bleeding point. Had this artery been originally opened and the omentum not protruded, no doubt she would have had concealed hemorrhage. Stab wounds of the abdomen are less frequently attended by lesions of the intestines than are gunshot wounds; the cutting instrument slips between the intestinal folds. Yet it is not safe to close up these wounds without search for such injuries.

You cannot rely on shock as a sign of intestinal wounding. It is more frequently absent than present.

CASE 5. *Infra-orbital Tic. Neurectomy, Complete Relief.*—Mrs. M. R., female, white, aged sixty-two years, housekeeper, was admitted to the private wards September 1, 1896, suffering with neuralgia of the right superior maxillary nerve, the second branch of the fifth pair of cranial nerves, of seventeen years' duration. Family and general history good. The disease has gradually increased in severity; beginning with occasional slight attacks at long intervals, it was now constant and so severe that she had resorted to the regular use of morphine for the past year and a half, taking six grains subcutaneously per diem.

September 2, 10 A.M., assisted by Dr. McHatton, Dr. Little, and Dr. Derry, the latter administering ether, I proceeded to resect the nerve in the floor of the orbit. A vertical incision was made along the side of the nose from the lachrymal tubercle down to the wing of the nostril. Another horizontal cut was made along the margin of the orbit, beginning nearly at the middle of the first incision. These cuts were from an inch to one and one half in length, and down to the bone. Bleeding vessels were caught, and the search for the nerve as it comes out of infra-orbital foramen was begun by dissecting up the lower flap. This was accomplished after some difficulty and a ligature put around it.

The upper flap was then raised together with the contents of the orbital fossa, exposing the floor of the orbit as far back as possible. The infra-orbital canal was easily located, and opened up with the edge of the knife. The nerve was lifted out and the dissection carried backwards across the spheno-maxillary fossa, nearly to the foramen rotundum where the nerve comes out of the cranial cavity. At this point the nerve was divided with its attendant artery, was drawn out and cut again at the point where it divides to supply the cheek, nose, lip, and lower eyelid. A piece one and a half inches long was thus removed. The distal end was turned down, the bleeding controlled, and the external wound closed and dressed antiseptically. Primary union taking place, she left the hospital September 8, seven days after the operation.

For the first twenty-four hours she had no pain whatever, then a little pain was felt in the inferior dental nerve as it leaves the mental foramen. So great was the relief from her suffering that she began at once cutting down her morphine habit: the day she left the hospital she took only half a grain, and since that time she has never taken it again. The pain in the inferior dental nerve continued for about seven weeks and then disappeared. I have purposely refrained from reporting this case until the present time because I wished to be sure of the relief for six or seven months.

Simple section of the nerve gives no permanent relief; if any it is only temporary, the pain returns with the reunion of the divided ends of the nerve. Resection of the nerve is followed by relief, sometimes lasting from one to three years, many times it is permanent. In the present instance I hope it will be permanent, as the cause of the trouble seems to have been disease of the nerve sheath, which was found to be thickened and inflamed.

If the trouble is central no operation will give relief; if located in the Gasserian ganglion perhaps recurrence will take place. If such should be the case, then the more radical operation, excision of the two lower sections of the ganglion in the cranial cavity, will have to be resorted to for relief.

In performing resection of nerves for inveterate neuralgia, folding back the distal end of the nerve trunk seems to enhance the value of the operation, while if the proximal end is bent back the neuralgia will redevelop. Weir Mitchell says a nerve tumor develops on the end of the proximal stump if it is folded back, causing the pain after operation.

CASE 6. *Cystic Adenoma of the Thyroid Gland. Enucleation.*—Mrs. W. B. P., female, white aged twenty-five years, housekeeper, was admitted November 10, 1896, to the private wards, having a tumor of the left body of the thyroid gland. Family and previous history good, married eight years, had one child. She first noticed the growth in the neck seven years ago. Its progress has been slow and painless, not giving her any discomfort except from its unsightliness. Recently it has interfered with deglutition and presses on the trachea. It extends from the top of the larynx down to the clavicle, is oval in shape, four inches broad and four and one-half inches long, and is semi-solid, fluctuating at one point.

November 11, 10 A. M., assisted by Dr. Derry, Dr. Weaver, and Dr. Little, the former giving chloroform, I proceeded to enucleate the tumor by making a longitudinal incision over the most prominent part of the growth in a line from the angle of the jaw to the sterno-clavicular articulation. The tissues were divided layer by layer, the overlying muscles (the sterno-hyoid, omo-hyoid, and sterno-thyroid) separated and pushed aside, all bleeding controlled until the gland was reached. The capsule of the gland and the gland tissues (a very thin layer surrounding the tumor) were divided down to the tumor, its capsule was found and the growth peeled out. Severe hemorrhage from the bed of the tumor followed its enucleation, the blood welling up so rapidly that the exact point, if any, of bleeding could not be located. The cavity was packed tightly with iodoform gauze and the edges of the wound brought together with catgut sutures, provisional sutures being left untied over the lower angle of the wound where the ends of the packing were left out. The wound was dressed and the patient put to bed with a nurse sitting by her side to watch for hemorrhage. At the end of forty-eight hours the dressings were removed, the packing withdrawn, and the provisional sutures tied. Primary union took place, and she left the hospital on the eighth day after the operation. The tumor had one large and several smaller cysts in its body, the rest was semi-solid.

Adenoma, as you know, is a benign epithelial tumor, which in structure resembles the glandular tissues in which the growth develops; in the thyroid, of course, it resembles thyroid tissue. Growths in this gland, if benign, are adenomata, except the ordinary bronchocele, which is not a tumor, but is a swelling of miasmatic or infective origin produced by the local action of an unknown microbe. Adenomata are distinctly encapsulated and are embedded in the substance of the gland affected; they receive a rich blood supply and grow to an enormous size, causing difficulty by pressure upon important parts. The tumors undergo gelatinous changes and cystic degeneration. Hence the mistake of considering these tumors, when fluctuating, simple cysts. The proper treatment is enucleation. Local applications of iodine, etc., and the internal use of iodine compounds have no effect whatever, while they are beneficial in bronchocele or miasmatic struma. Extirpation of the entire gland, or portions of it, are not needed in thyro-adenoma; complete extirpation alone should be reserved for the malignant growths of the gland; it is folly to expose a patient to myxedema or cretinism, the result of complete destruction of this body in benign growths. Partial extirpation is rarely ever indicated, but can be performed without the resulting myxedema.

Fortunately for the patient she had refused various procedures directed to letting out the fluid contents of the growth, aspiration, electrolysis, etc. One gentleman, after she had consented to go into the hospital for operation, urged her to let him thrust a trocar into the growth and let the fluid out, saying she did not need a cutting operation. Treatment of this kind complicates enucleation by provoking inflammations and adhesions, making the steps of the operation, which are simple, difficult.

---

### TEST FOR SUGAR IN THE URINE.

The *New York Medical Times* gives the following test for sugar in the urine: Add to a small quantity of urine the same amount of saturated solution of picric acid. To this add half as much more liquor potass. If sugar is present the color becomes very dark and opaque. If no sugar is present the color is a handsome bright red.—*Ex.*

